# Comprehensive analysis of efferocytosis-related genes in diagnosis and immune infiltration in atherosclerosis: based on bulk and single-cell RNA sequencing data

**DOI:** 10.3389/fcell.2026.1838380

**Published:** 2026-05-26

**Authors:** Jintao Qian, Junhao Zhang, Jiahui Wang, Suyu Xu, Li Yu, Chuiyu Kong, Qing Zhou

**Affiliations:** 1 Department of Cardiothoracic Surgery, Nanjing Drum Tower Hospital, Affiliated Hospital of Medical School, Nanjing University, Nanjing, China; 2 Department of Cardiothoracic Surgery, Nanjing Drum Tower Hospital Clinical College of Nanjing University of Chinese Medicine, Nanjing, China; 3 School of Pharmacy, Nanjing University of Chinese Medicine, Nanjing, China

**Keywords:** atherosclerosis, biomarkers, efferocytosis, machine learning, single-cell

## Abstract

**Background:**

Atherosclerosis (AS) is a widespread cardiovascular disorder that constitutes a major contributor to global morbidity and mortality, thereby imposing significant economic burdens on healthcare systems worldwide. Efferocytosis, the phagocytic removal of apoptotic cells, serves as a fundamental mechanism for maintaining tissue homeostasis during normal physiological function and for restoring equilibrium following pathological insults.

**Methods:**

This study systematically investigated the functional roles of efferocytosis across specific cell types using single-cell datasets. By integrating differential expression analysis, weighted gene co-expression network analysis (WGCNA), and machine learning approaches, six key genes were identified. Gene set variation analysis (GSVA) was subsequently performed to elucidate the biological pathways in which these genes are involved. Furthermore, the ssGSEA algorithm was applied to assess the association between these genes and immune cell infiltration levels. To evaluate their diagnostic potential, a nomogram was constructed based on the gene signature. Unsupervised consensus clustering revealed two distinct molecular subtypes of atherosclerosis. Finally, the protein expression levels of core EFRGs in atherosclerosis were analyzed using Western blotting.

**Results:**

Single-cell data analysis demonstrates that macrophages, vascular smooth muscle cells, and endothelial cells play potential functional roles in efferocytosis. Utilizing bulk RNA sequencing, six core efferocytosis-related genes (STAB1, ANO5, GULP1, LGR6, SCARF1, and CAMK2G) were identified, which exhibit significant diagnostic potential in atherosclerosis. Based on the expression profiles of these six genes, atherosclerosis can be stratified into two distinct molecular subtypes—subtypes A and B—with subtype B being characterized by its association with unstable plaque formation. Western blot analysis confirmed the expression trends of five proteins (ANO5, GULP1, LGR6, SCARF1, and CAMK2G) among the candidates.

**Conclusion:**

This study has revealed the potential value of efferocytosis-related biomarkers in the diagnosis of atherosclerosis (AS) and the optimization of treatment strategies, providing new theoretical basis and research perspectives for precise intervention in cardiovascular diseases.

## 1 Introduction

Atherosclerosis (AS) is a chronic inflammatory disorder characterized by the pathological accumulation of lipids, inflammatory cells, and fibrous components within the arterial intima, culminating in the formation of atherosclerotic plaques (Dabravolski et al., 2025). As a major underlying pathology of cardiovascular diseases, AS continues to be a leading cause of global morbidity and mortality (Libby, 2021). The economic impact of AS is substantial, largely attributed to its severe clinical sequelae, such as myocardial infarction and stroke (Davies, 1996; Kotlyarov, 2024). Contemporary management approaches primarily involve lifestyle interventions, pharmacological treatments including statin therapy, and surgical procedures such as percutaneous coronary angioplasty (Khera et al., 2016; Wang et al., 2024). Nevertheless, these conventional strategies exhibit variable therapeutic efficacy and are often associated with adverse effects, highlighting the critical need for novel diagnostic tools and targeted therapeutic approaches that can enhance clinical outcomes in patients with AS.

Efferocytosis, defined as the phagocytic clearance of apoptotic cells by both professional and non-professional phagocytes, plays a critical role in the maintenance of tissue homeostasis (Doran et al., 2020). Recent studies have increasingly emphasized the role of efferocytosis in the pathophysiological mechanisms underlying atherosclerosis (Adkar and Leeper, 2024). Accumulating evidence demonstrates that efficient efferocytosis modulates inflammatory responses and contributes to the structural stability of atherosclerotic plaques (Doddapattar et al., 2022). Defective efferocytosis has been linked to persistent inflammation and accelerated plaque progression, suggesting that therapeutic enhancement of this process may offer a promising strategy for mitigating atherosclerosis (AS). In recent years, therapeutic strategies that target and regulate specific functional genes to intervene in the occurrence and development of atherosclerosis have become an important Frontier direction in cardiovascular basic and translational research (Min et al., 2024). Under this background, systematically analyzing the dynamic expression characteristics of efferocytosis-related genes (EFRGs) in atherosclerotic lesions may potentially promote their transformation into novel diagnostic markers and precise intervention targets with clinical application potential. To address these knowledge gaps, this study utilizes comprehensive bioinformatics approaches to analyze multiple gene expression datasets, with a focus on single-cell RNA sequencing (scRNA-seq) data. This analytical framework enables the systematic investigation of cellular heterogeneity and facilitates the identification of distinct gene expression signatures associated with disease phenotypes. One of the key advantages of this proposed method lies in its ability to integrate large-scale multi-omics datasets, which helps us gain a deeper understanding of efferocytosis in atherosclerosis.

In summary, investigating efferocytosis within the context of atherosclerosis presents a promising strategy for discovering novel biomarkers and therapeutic targets. This study employed cutting-edge bioinformatics methods to systematically analyze the gene expression profiles of atherosclerotic tissues and control samples. The aim was to precisely identify the core differentially expressed genes (EFRGs) related to efferocytosis, and to elucidate their potential regulatory networks and functional enrichment characteristics. This would provide a preliminary direction for the development of targeted intervention strategies and the construction of personalized treatment plans.

## 2 Materials and methods

### 2.1 Data acquisition and human samples

The datasets used in this investigation were retrieved from the Gene Expression Omnibus (GEO) database (https://www.ncbi.nlm.nih.gov/geo/). The GSE100927 dataset included 69 patients with Atherosclerosis (AS) and 35 healthy control subjects (Steenman et al., 2018). The GSE43292 dataset served as the validation dataset in this study, which included 34 atherosclerotic plaque samples and 34 macroscopically normal tissue specimens (Ayari and Bricca, 2013). For bulk RNA-seq data, we used the ComBat function in the limma package to jointly correct the expression matrices of the training and validation sets. This effectively eliminated batch-related bias while preserving biologically meaningful variation. Importantly, to ensure the rigor of external validation and the reliability of our conclusions, all modeling procedures—including feature selection, model training, and hyperparameter tuning—were strictly confined to the training set; the validation set was never used during model development and served exclusively for final performance evaluation. The single-cell dataset GSE159677, derived from the proximal adjacent and atherosclerotic core regions of arterial plaques obtained from three patients (Alsaigh et al., 2022). For single-cell RNA-seq data analysis, we performed standard preprocessing steps, including library size normalization, log-transformation (using log1p), and identification of highly variable genes—standard practices to mitigate technical variation. A list of 272 genes associated with efferocytosis was obtained from a prior study (Cao et al., 2024). Three aortic tissue samples exhibiting visible atherosclerotic plaques and three morphologically intact aortic tissues were randomly selected from patients who underwent aortic replacement surgery due to aortic dilation at our institution. All human aortic samples (n = 6) were processed in accordance with protocols approved by the Ethics Committee of Nanjing Drum Tower Hospital. The tissues were obtained from Nanjing Drum Tower Hospital, and all experimental procedures involving human samples were conducted in compliance with the approved ethical guidelines (approval number: 2022-157-01). Written informed consent was obtained from all participants prior to their inclusion in the study.

### 2.2 Single-cell analysis of the efferocytosis phenotype in atherosclerosis (AS)

The single-cell dataset GSE159677 was obtained and processed for analysis using the Seurat and harmony packages (Butler et al., 2018). The quality control of single-cell RNA sequencing data begins by calculating the total UMI count (nCount_RNA), the number of detected genes (nFeature_RNA), the complexity of the transcriptome (log10GenesPerUMI), and the proportion of mitochondrial genes (mitoRatio) for each cell. Subsequently, cells are filtered and retained if their nCount_RNA is between 200 and 5,000, nFeature_RNA is greater than 200, log10GenesPerUMI is greater than 0.8, and mitoRatio is less than 0.2. At the same time, genes with an expression count of less than 100 are excluded. Then, scDblFinder is used to identify and remove double cells based on sample origin (Germain et al., 2021). Finally, SCTransform is used to regress and correct the cell cycle scores (S.Score, G2M.Score, CC. Difference) and mitochondrial proportion, resulting in a high-quality expression matrix suitable for downstream analysis. Subsequently, The AUCell package was employed to compute enrichment scores based on the area under the curve (AUC) for a predefined set of 272 efferocytosis-related genes. The “monocle” package was employed to perform developmental trajectory analysis based on genes exhibiting high dispersion and expression levels (Qiu et al., 2017). The R package “CellChat” was applied to analyze cellular communication networks and the associated molecular mechanisms across individual cells (Fang et al., 2022).

### 2.3 Identification of differentially expressed genes associated with efferocytosis in AS

After completing the data processing, differentially expressed genes were identified using the “limma” package with thresholds of |logFC| > 0.585 and an adjusted p-value (FDR) < 0.05, where FDR correction was performed using the Benjamini–Hochberg method. To illustrate the outcomes of this analysis, volcano plots and heatmaps were created using the R packages “ggplot2” and “pheatmap”, respectively. Subsequently, a Venn diagram analysis was conducted to determine the overlap between differentially expressed genes and efferocytosis-related genes (EFRGs), leading to the identification of a final subset of differentially expressed efferocytosis-related genes. The protein-protein interaction (PPI) network was constructed based on data retrieved from the STRING database (https://cn.string-db.org/).

### 2.4 Functional and pathway enrichment analysis

To systematically explore the functional roles of differentially expressed EFRGs, Gene Ontology (GO) and Kyoto Encyclopedia of Genes and Genomes (KEGG) enrichment analyses were conducted using the “clusterProfiler” R package (Yu et al., 2012). Furthermore, gene set enrichment analysis (GSEA) was employed to investigate the potential biological roles of differentially expressed genes by examining their correlations across the full transcriptomic profile (Liberzon et al., 2015). Simultaneously, gene set variation analysis (GSVA) was conducted to assess alterations in pathway activity among individual samples, thereby revealing functional differences between experimental groups (Hänzelmann et al., 2013).

### 2.5 Identification of critical gene modules involved in AS

Optimization of the weighted gene co-expression network analysis (WGCNA) was carried out to detect functionally significant gene modules linked to atherosclerosis (AS) (Ravasz et al., 2002). We set the minimum module size to 50 genes and applied a dynamic tree cut with a merge cutoff of 0.25. To explore the connections between these modules and clinical characteristics, Pearson correlation analysis was conducted, leading to the identification of the module with the strongest correlation for subsequent in-depth study.

### 2.6 Machine learning-based identification of efferocytosis-associated diagnostic biomarkers

We utilized three machine learning algorithms—specifically, Least Absolute Shrinkage and Selection Operator (LASSO) regression, support vector machine (SVM), and random forest (RF)—to identify the core EFRGs associated with AS. The LASSO regression was conducted using the “glmnet” package to analyze the preprocessed data, from which key parameters such as lambda values, likelihood values, and classification error rates were derived. Subsequently, the results were visualized to facilitate interpretation. Concurrently, the “e1071” package was utilized to perform Support Vector Machine (SVM) analysis on the dataset, generating model prediction results, which were subsequently visualized for enhanced interpretability. The random forest algorithm was implemented using the “randomForest” package to perform a comprehensive analysis on the dataset, extract variable importance scores, and visualize the results for enhanced interpretability.

### 2.7 Gene expression validation and nomogram model development

The Wilcoxon rank-sum test was employed to assess differences in gene expression levels between the disease group and the control group. Receiver operating characteristic (ROC) curves were generated using the “pROC” R package. The Nomogram-related model was developed using the “rms” package, and its visualization was subsequently generated to facilitate intuitive interpretation.

### 2.8 Analysis of immune infiltration

Utilizing the ssGSEA algorithm implemented in the R software package “GSVA”, we evaluated immune cell infiltration in the dataset by incorporating 28 immune cell markers previously reported in the literature (Charoentong et al., 2017). The Wilcoxon rank-sum test was applied to evaluate the differences in immune cell infiltration scores between the two groups. Spearman’s correlation analysis was further conducted to assess the association between these infiltration scores and the core EFRGs within the disease group.

### 2.9 Identification of the efferocytosis subtypes associated with atherosclerosis

To examine potential subtypes of efferocytosis in atherosclerosis (AS), unsupervised consensus clustering analysis was conducted with the “ConsensusClusterPlus” R package, utilizing the expression signatures of EFRGs (Wilkerson and Hayes, 2010). The analytical framework applied the following parameters: a maximum of 5 clusters, the PAM clustering algorithm, Spearman correlation as the distance metric, and 100 iterations to improve the robustness of the results. According to the clustering results, the AS samples were divided into two distinct subtypes of efferocytosis, designated as subtype A and subtype B, corresponding to Cluster 1 and Cluster 2, respectively. The distribution patterns of the two sample groups are visualized using principal component analysis (PCA).

### 2.10 Western blot

Total protein was extracted from human aortic tissue using RIPA lysis buffer (Beyotime, China, #P0013B) supplemented with 1% protease inhibitor cocktail (cOmplete™ Protease Inhibitor Cocktail, Roche). Protein concentration was quantified using the BCA protein assay kit (Beyotime, China, #P0010). Protein samples were mixed with 5× SDS-PAGE loading buffer (Beyotime, China, #P0015L), denatured by heating at 100 °C for 10 min, and immediately cooled on ice. Denatured proteins were separated by SDS-PAGE on a YoungPAGE™ Bis-Tris gel (4%–20%, 10 cm × 8 cm, 15 wells) and transferred onto a PVDF membrane (Millipore, Burlington, MA, United States). The membrane was blocked with TBST buffer containing 5% skim milk at room temperature for 1 h. Following blocking, the membrane was incubated overnight at 4 °C with primary antibodies diluted in 5% BSA. The primary antibodies used were: Anti-LGR6 (1:500, rabbit monoclonal, #CY8621, Abways, China), Anti-CaMK2G (1:500, rabbit polyclonal, #CY7228, Abways, China), Anti-ANO5 (1:500, rabbit polyclonal, #YN3927, Immunoway, China), Anti-SCARF1 (1:500, rabbit polyclonal, #YN2393, Immunoway, China), Anti-GULP1 (1:500, rabbit polyclonal, #YN7965, Immunoway, China), Anti-STAB1 (1:500, rabbit polyclonal, #bs-7510r, Bioss, China), Anti-GAPDH (1:1,000, rabbit monoclonal, #AF0006, Beyotime, China), and Anti-β-actin (1:1,000, mouse monoclonal, #AA128, Beyotime, China). After three washes with TBST, the membrane was incubated with HRP-conjugated goat anti-rabbit or anti-mouse IgG secondary antibodies (1:2,000) at 37 °C for 1 h. Following extensive washing, the membrane was developed using ECL chemiluminescent substrate (Vazyme, China), and the signals were detected using a ChemiDoc™ imaging system (Bio-Rad, CA, United States).

### 2.11 Statistical analysis

All statistical analyses of bioinformatics-related data were carried out using R software (version 4.3.3), and data visualization was performed using the ggplot2 package. Western blot data were analyzed with GraphPad Prism 7.0 (United States). Experimental results are expressed as mean ± standard deviation (Mean ± SD). Group differences were assessed using the unpaired Student’s t-test. A *P* value <0.05 was considered statistically significant.

## 3 Results

### 3.1 Investigation of the efferocytosis phenotype in atherosclerosis-related single-cell data

**FIGURE 1:**
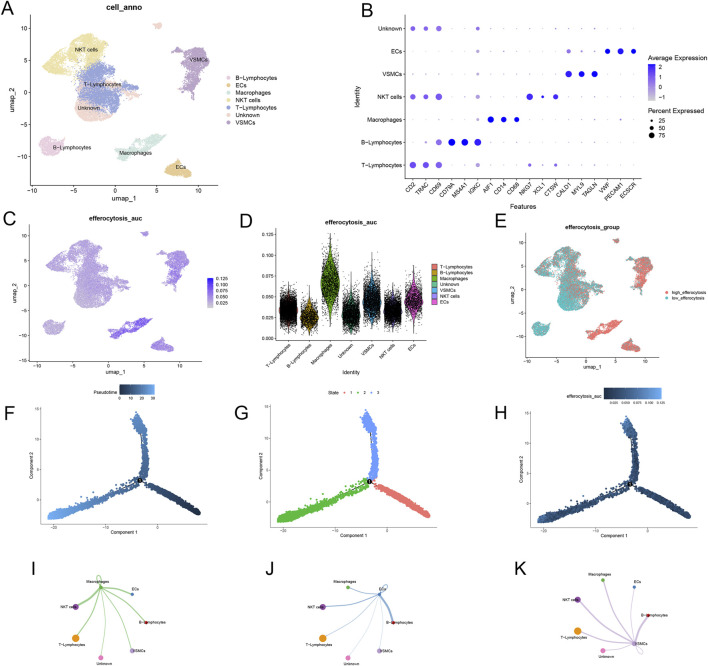
Single-cell transcriptomic analysis of AS. **(A)** UMAP plot illustrating six major cell types. **(B)** Dot plot depicting cell type-specific marker genes. **(C,D)** UMAP and violin plots illustrating the distribution of cells according to AUC values; darker color intensity corresponds to higher AUC values. **(E)** The UMAP plot shows the groups with low and high AUC values. The red dots represent the group with high Efferocytosis AUC score, while the blue dots represent the group with low Efferocytosis AUC score. **(F)** The pseudo-time trajectory is presented in the form of color gradation, with the color gradually changing from dark blue to light blue, reflecting the continuous evolutionary process of the cells along the proposed time sequence. **(G)** The pseudotime trajectory has been segmented into three distinct states through the application of Monocle. **(H)** Pseudotime trajectory constructed using AUCell scores, where blue hues represent elevated efferocytosis activity. **(I–K)** This network diagram is based on the analysis results of CellChat and visually presents the intercellular communication relationships between macrophages, endothelial cells (ECs), vascular smooth muscle cells (VSMCs) and other cell types. The thickness of the lines is positively correlated with the intensity of the interaction.

As illustrated in Figure 1A, cell classification was performed according to the marker genes specifically expressed by each cell type described in the original study. The cells that could not be definitively assigned to any of the six main subgroups were categorized as “unknown” (Alsaigh et al., 2022). The six primary cell types identified in this study are as follows: endothelial cells (ECs), vascular smooth muscle cells (VSMCs), B lymphocytes, T lymphocytes, natural killer T (NKT) cells, and macrophages. The expression patterns of marker genes for each cell type were illustrated via dot plots (Figure 1B). Figures 1C,D illustrate the distribution of AUC values corresponding to different cell populations in the enrichment analysis of genes related to efferocytosis; among them, macrophages show the highest AUC score. Figure 1E shows the distribution of cell populations stratified into high- and low-efferocytosis groups based on median AUCell AUC values associated with efferocytosis. The results show that the high-efferocytosis group was predominantly enriched in macrophages, endothelial cells, and vascular smooth muscle cells. We developed a pseudotime trajectory of cellular progression (Figure 1F) to investigate the dynamic regulatory mechanisms and gene expression profiles involved in efferocytosis. Notably, the transitional phases along the trajectory demonstrate distinct developmental patterns (Figure 1G). Subsequently, we illustrated the variations in AUCell scores across the entire evolutionary trajectory, with State 3 displaying significantly enhanced efferocytosis activity (Figure 1H). Finally, we illustrated the intercellular communication networks involving three cell types with elevated efferocytosis AUC values—endothelial cells (ECs), vascular smooth muscle cells (VSMCs), and macrophages—in interaction with other cellular components (Figures 1I–K).

### 3.2 Identification and functional analysis of differentially expressed genes linked to efferocytosis in AS

**FIGURE 2:**
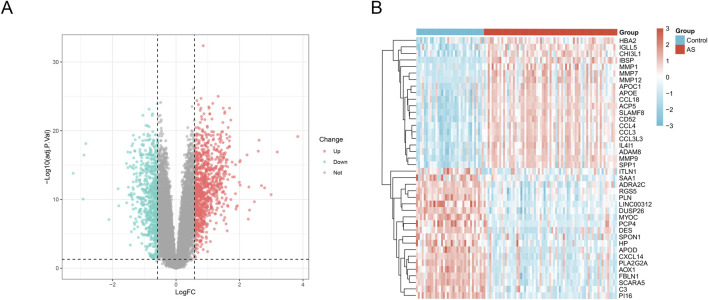
Identification of differentially expressed genes in atherosclerosis. **(A)** Volcano plot illustrating differentially expressed genes between atherosclerosis and control groups. Here, red nodes represent upregulated genes, while blue nodes represent downregulated genes. **(B)** Heatmap illustrating the expression patterns of the top 20 significantly upregulated and downregulated differentially expressed genes (DEGs) between the AS and control groups. The color gradient ranges from blue to red, reflecting the increasing expression levels of the genes.

**FIGURE 3:**
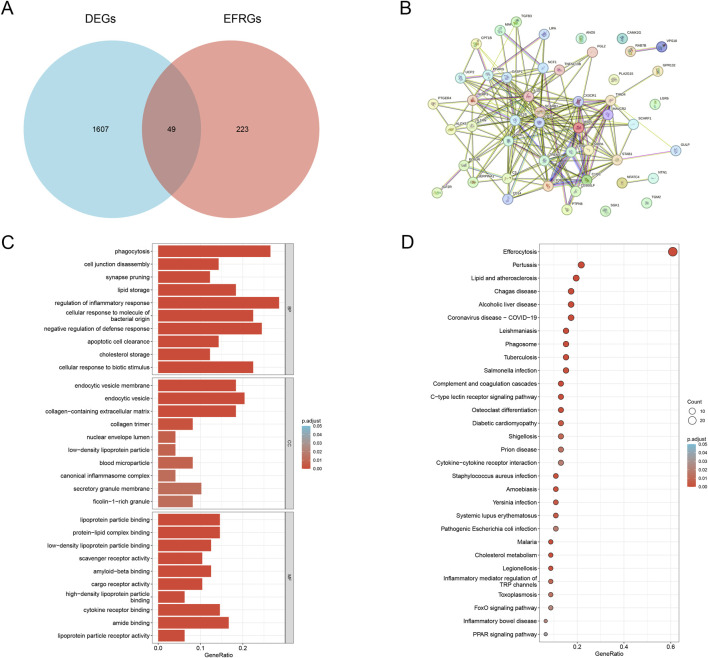
Identification and functional enrichment analysis for AS-EFRGs. **(A)** Venn diagram showing the overlap between DEGs and EFRGs, identifying 49 AS-EFRGs. **(B)** Protein-protein interaction analysis of 49 AS-EFRGs. **(C)** GO enrichment analysis of 49 AS-EFRGs. The horizontal axis represents the gene ratio (GeneRatio), and the color indicates the adjusted p-value after multiple tests (adjusted p-value). **(D)** KEGG enrichment analysis of 49 AS-EFRGs. The horizontal axis represents the gene ratio (GeneRatio), and the color coding reflects the adjusted p-value after multiple tests (adjusted p-value). The area of the circle is proportional to the number of enriched genes.

We performed a differential gene expression analysis on the GSE100927 dataset using the selection criteria of |logFC| > 0.585 and adjusted p-value (FDR) < 0.05, identifying a total of 990 upregulated genes and 666 downregulated genes (Figure 2A). Subsequently, we visualized the expression profiles of the top 20 upregulated and top 20 downregulated genes with the highest absolute logFC values using a heatmap (Figure 2B). Subsequently, we performed an intersection analysis between the 1,656 differentially expressed genes and the 272 genes associated with efferocytosis, ultimately identifying 49 atherosclerosis-associated efferocytosis-related genes (AS-EFRGs) (Figure 3A). A protein-protein interaction (PPI) network was constructed based on these 49 genes (Figure 3B). In the subsequent Gene Ontology (GO) enrichment analysis of these 49 genes, we observed significant enrichment in biological processes (BP), such as “regulation of inflammatory response”, cellular components (CC), such as “endocytic vesicle”, and molecular functions (MF), such as “lipoprotein particle binding” (Figure 3C). KEGG pathway enrichment analysis further indicated that these genes were enriched in pathways associated with “efferocytosis” (Figure 3D).

### 3.3 WGCNA of gene modules associated with AS

**FIGURE 4:**
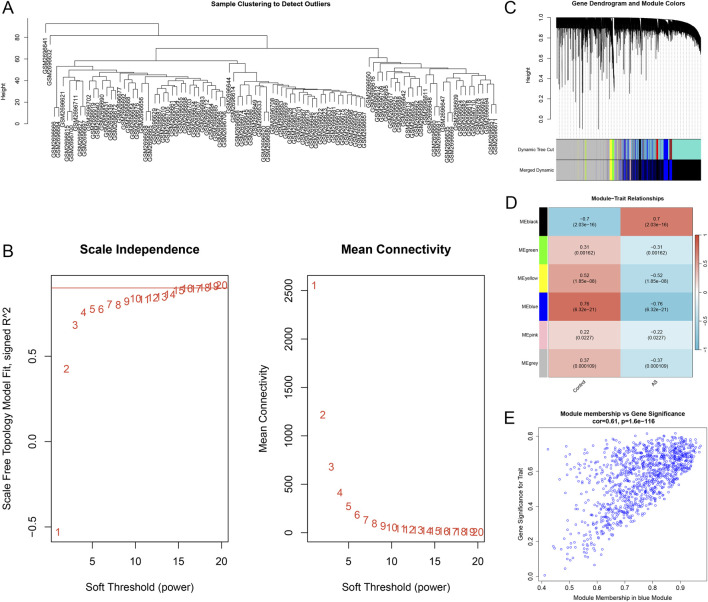
WGCNA identifies significant gene modules associated with AS. **(A)** Clustering analysis of samples for outlier detection. **(B)** Scale-free topology analysis for determining the optimal soft threshold. **(C)** Gene dendrogram showing modules identified through dynamic tree cutting. **(D)** Carried out Spearman correlation analysis between the gene modules and clinical characteristics; in the figure, blue indicates negative correlation, red indicates positive correlation, and the saturation of the color (i.e., the shade of the hue) reflects the absolute value of the correlation coefficient. **(E)** Scatter plot demonstrating the association between module membership and gene significance in the blue module.

To identify modular gene clusters associated with atherosclerosis (AS), we performed weighted gene co-expression network analysis (WGCNA). Preliminary clustering analysis of the 104 samples was carried out, and no significant outlier samples were identified during the process (Figure 4A). Ultimately, a soft threshold value of β = 18 was determined the establishment of the co-expression network (Figure 4B). Gene clustering was performed through the dynamic tree cutting technique, and modules sharing comparable features were subsequently merged (Figure 4C). Further analysis revealed correlations between the six co-expression modules identified through partitioning and both the control group and the AS group. Among these, the blue module exhibited the most significant association with AS (r = −0.76, P = 6.32 × 10^−21^) (Figure 4D). Finally, a scatter plot was constructed to illustrate the relationship between gene significance and module membership for the blue module. The analysis revealed a statistically significant positive correlation (r = 0.61, p < 1.6 × 10^−16^), further supporting the hypothesis that the collective effect of genes within this module plays a critical role in disease association (Figure 4E). The genes within the blue module were selected as the candidate target gene set for subsequent bioinformatics analysis.

### 3.4 Identification of characteristic EFRGs biomarkers for AS

**FIGURE 5:**
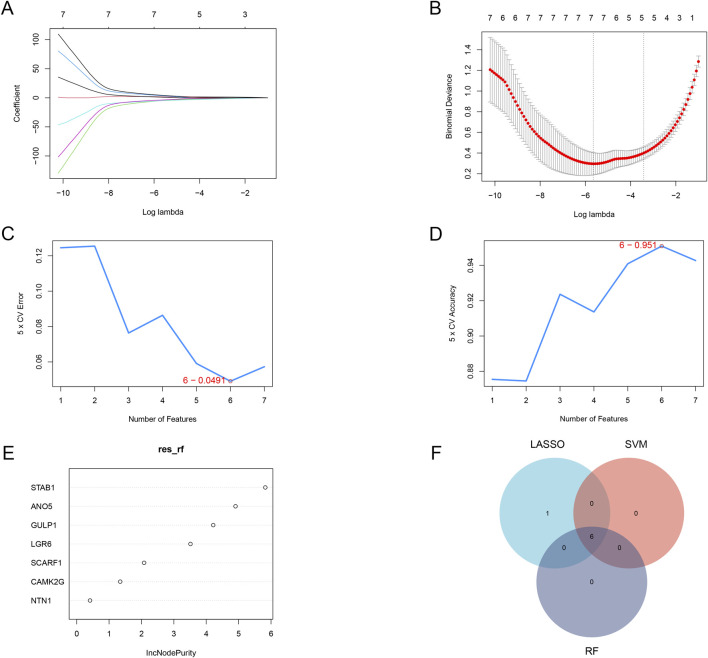
Identification of characteristic EFRGS associated with AS. **(A,B)** Variable selection using the LASSO algorithm included coefficient plots and partial likelihood deviation. **(C,D)** The SVM model used 10-fold cross-validation to show accuracy and error trends. **(E)** Variable importance among the 7 key AS-EFRGs was ranked using the Random Forest algorithm. **(F)** Venn diagram illustrating the six common genes identified by three machine learning techniques.

The blue module genes identified through WGCNA analysis were intersected with a previously screened set of 49 differentially expressed genes associated with efferocytosis, leading to the identification of 7 overlapping candidate genes. For these seven genes, we applied three machine learning algorithms—LASSO, SVM, and RF—to perform feature selection and model optimization. Following Lasso algorithm-based filtering, seven genes were ultimately selected (Figures 5A,B). Through SVM-based screening, six genes were identified (Figures 5C,D). Similarly, application of the RF algorithm resulted in the identification of six genes (Figure 5E). Ultimately, the intersection of genes selected by the three algorithms identified six biomarkers—STAB1, ANO5, GULP1, LGR6, SCARF1, and CAMK2G—for atherosclerosis (Figure 5F).

### 3.5 Evaluation of the diagnostic performance of efferocytosis-related biomarkers

**FIGURE 6:**
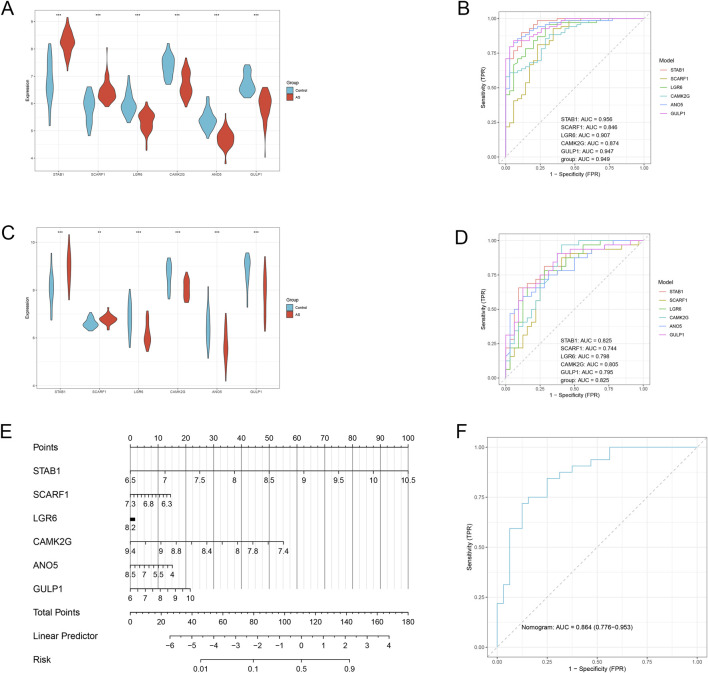
Diagnostic potential of efferocytosis-related biomarkers in AS. **(A,B)** Expression profiles and ROC curves of the six genes in the GSE100927. **(C,D)** Expression profiles and ROC curves of the six genes in the GSE43292. **(E)** Nomogram model constructed using the six-gene signature. **(F)** ROC curve of the nomogram model.

To validate the reliability of the six core biomarkers, we further examined their expression differences in the training cohort GSE100927. The results demonstrated that all six genes exhibited statistically significant differential expression. Moreover, ROC curve analysis revealed AUC values exceeding 0.7 for each gene (Figures 6A,B). These findings were corroborated in the independent validation cohort GSE43292, where all six biomarkers showed consistent differential expression and AUC values greater than 0.7 (Figures 6C,D). Based on these six genes, we developed a diagnostic nomogram using the independent external validation cohort GSE43292, achieving an area under the ROC curve (AUC) of 0.864 (Figures 6E,F).

### 3.6 Gene set variation analysis of EFRGs

**FIGURE 7:**
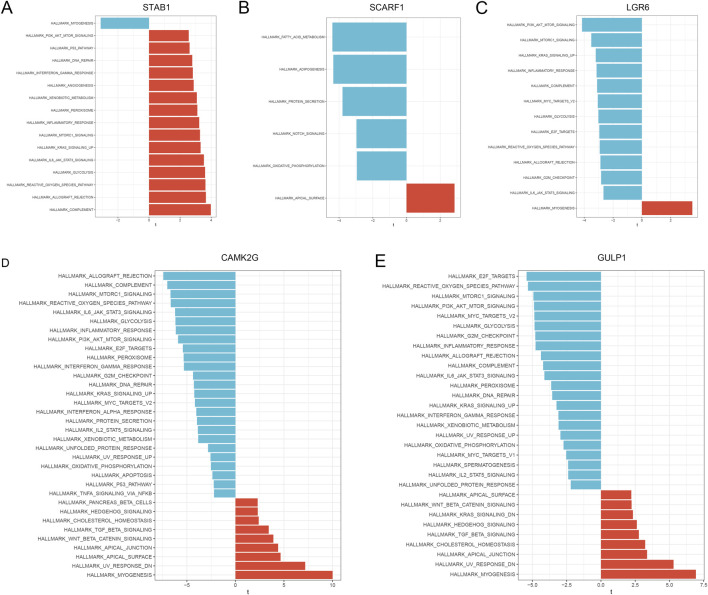
GSVA analysis of the six EFRGs. Signaling pathways linked to **(A)** STAB1, **(B)** SCARF1, **(C)** LGR6, **(D)** CAMK2G, and **(E)** GULP1 (P adj <0.05). Red bars illustrate pathways enriched in the high-expression group, whereas blue bars highlight those in the low-expression group.

We divided the disease group into high-expression subgroups and low-expression subgroups based on the average expression level of EFRGs, and conducted GSVA enrichment analysis based on this grouping. The pathway enrichment results of STAB1, SCARF1, LGR6, CAMK2G, and GULP1 were all selected after multiple test correction (FDR <0.05); ANO5 did not show statistically significant pathway enrichment (Figures 7A–E). In this study, elevated expression of STAB1 was positively correlated with the “COMPLEMENT” and “ALLOGRAFT REJECTION” pathways, whereas negative enrichment was observed in the “MYOGENESIS” pathway. Similarly, high expression of SCARF1 demonstrated significant positive enrichment in the “APICAL SURFACE” pathway, but exhibited negative enrichment in the “FATTY ACID METABOLISM” and “ADIPOGENESIS” pathways. LGR6 showed marked positive enrichment within the “MYOGENESIS” pathway, while displaying negative enrichment in both the “PI3K AKT MTOR SIGNALING” and “MTORC1 SIGNALING” pathways. CAMK2G and GULP1 also exhibited significant positive enrichment in the “MYOGENESIS” and “UV RESPONSE DN” pathways, yet were negatively enriched in the “ALLOGRAFT REJECTION”/“COMPLEMENT” (CAMK2G) and “E2F TARGETS”/“REACTIVE OXYGEN SPECIES PATHWAY” (GULP1) pathways, respectively.

### 3.7 Analysis of the association between immune infiltration and AS-EFRGs

**FIGURE 8:**
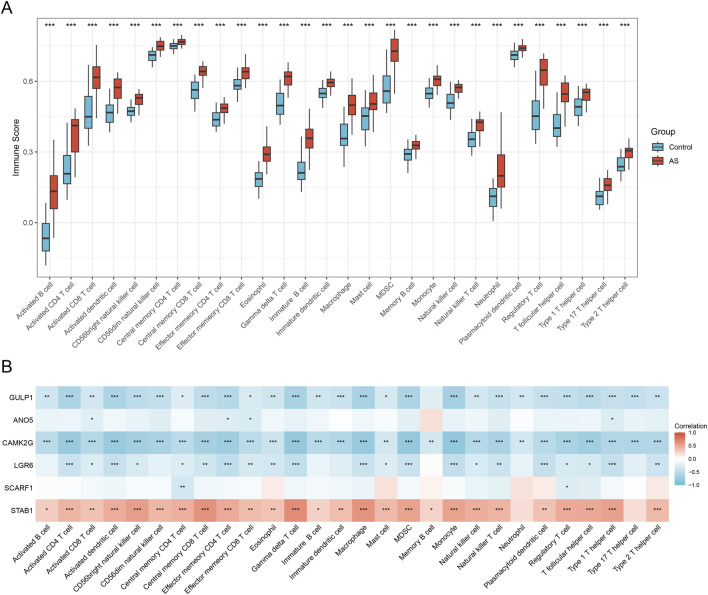
Immune infiltration analysis and its correlation with EFRGs in AS. **(A)** ssGSEA analysis of 28 immune cell types in control and AS groups. **(B)** Spearman correlation analysis between the six EFRGs and 28 types of immune cells (P < 0.05; **P < 0.01; ***P < 0.001); in the figure, blue indicates negative correlation, red indicates positive correlation, and the color saturation reflects the absolute value of the correlation coefficient.

We performed ssGSEA analysis on the GSE100927 dataset using 28 immune cell-specific marker genes identified in previous studies. The results demonstrated that nearly all types of immune cells exhibited statistically significant differences between the control group and the AS group (Figure 8A). Subsequently, we conducted further analysis of the correlations between the six core genes and 28 types of immune cells. The results indicated that STAB1 exhibited a significantly positive correlation with almost all immune cell types, whereas GULP1, CAMK2G, and LGR6 predominantly showed negative correlations with most immune cells. In contrast, no statistically significant correlations were identified between SCARF1 and ANO5 and the majority of immune cell types (Figure 8B).

### 3.8 Identification of molecular signatures associated with efferocytosis

**FIGURE 9:**
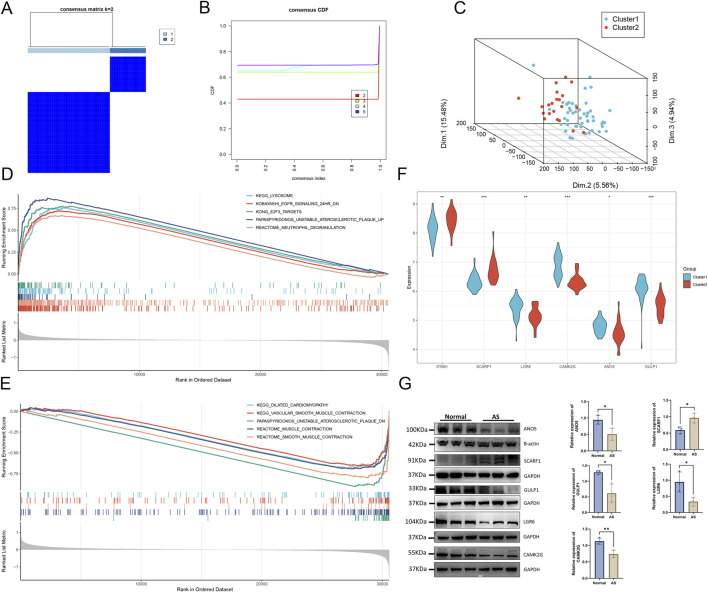
Analysis of efferocytosis-related molecular patterns in AS. **(A)** Consensus clustering matrix when k equals 2. **(B)** CDF curves for k values ranging from 2 to 5. **(C)** The PCA scatter plot shows the distribution of each sample in the principal component space; among them, the blue dots represent Cluster 1, and the red dots represent Cluster 2. GSEA pathways that are enriched in subtype A **(D)** and subtype B **(E)**, respectively. **(F)** Expression profiles of the six genes in the two distinct subtypes. **(G)** Western blot analysis of the expression levels of six core EFRGs in aortic tissues from individuals with and without AS (n = 6) (*P < 0.05; **P < 0.01; ***P < 0.001).

To elucidate the molecular characteristics of efferocytosis in atherosclerosis (AS), consensus clustering analysis was conducted on samples obtained from the AS patient cohort. When k = 2, the samples were distinctly partitioned into two independent clusters (Figure 9A). Figure 9B illustrates the cumulative distribution function (CDF) curves across varying k values. Principal Component Analysis (PCA) further confirmed the significant differences between the two subtypes. We defined cluster1 and cluster2 as subtype A and subtype B, respectively (Figure 9C). Subsequently, GSEA enrichment analysis was performed on both subtype A and subtype B, revealing five pathways that were significantly positively enriched and five pathways that were significantly negatively enriched in subtype B (Figures 9D,E). We focused on the “PAPASPYRIDONOS UNSTABLE ATEROSCLEROTIC PLAQUE UP” pathway identified through positive enrichment analysis and the “PAPASPYRIDONOS UNSTABLE ATEROSCLEROTIC PLAQUE DN” pathway identified through negative enrichment analysis. Based on the pathway information derived from enrichment analysis, we classified subtype B as a high-risk subgroup. Finally, the expression patterns of six EFRGs in subgroups A and B were further analyzed, and the observed trends were consistent with those between the control group and the AS group (Figure 9F).

### 3.9 Protein-level validation of core EFRG expression

Western blot analysis demonstrated that, compared with the normal control group, the expression levels of ANO5, GULP1, LGR6, and CaMK2G proteins were significantly reduced in the atherosclerosis group (*p* <0.05), whereas SCARF1 expression was markedly upregulated (*p* <0.05). These findings were consistent with those of the previous analysis. No statistically significant difference was observed in the expression of STAB1 protein between the two groups.

## 4 Discussion

The pathophysiology of atherosclerosis (AS) is characterized by intricate interactions among diverse cellular and molecular elements, encompassing dysregulated lipid metabolism, endothelial dysfunction, and sustained inflammatory responses (Fan and Watanabe, 2022). The economic impact of AS on healthcare systems is considerable, given its association with severe clinical complications such as myocardial infarction and stroke (Bułdak, 2022). These consequences highlight the urgent need for novel diagnostic and therapeutic approaches that can effectively modulate disease progression. This study aims to investigate the efferocytosis phenotype in atherosclerosis and evaluate its potential as a therapeutic target. Accumulating evidence suggests that impaired efferocytosis plays a critical role in promoting atherosclerotic plaque progression and chronic inflammation (Yurdagul et al., 2020). By integrating advanced bioinformatics methodologies with machine learning techniques, we sought to identify differentially expressed efferocytosis-related genes (EFRGs) and elucidate their functional roles in atherosclerosis (AS), thereby contributing to the discovery of novel diagnostic biomarkers and targeted therapeutic strategies. The following discussion will systematically elaborate on the core findings of this study and objectively evaluate the potential translational value of the identified biomarkers in clinical diagnosis, risk stratification, and targeted intervention.

In this study, we first identified three cell types closely associated with efferocytosis through single-cell transcriptomic analysis—macrophages, vascular smooth muscle cells, and endothelial cells—and further investigated the dynamic alterations of efferocytosis scores within pseudo-temporal trajectories. Subsequently, using conventional transcriptomic datasets, we performed differential expression analysis, weighted gene co-expression network analysis (WGCNA), and applied three distinct machine learning algorithms. By integrating these complementary bioinformatics methods, we identified six efferocytosis-related genes (EFRGs) that may play a significant role in atherosclerosis: STAB1, ANO5, GULP1, LGR6, SCARF1, and CAMK2G. Based on these core genes, we developed a diagnostic nomogram model, which demonstrated exceptional discriminative ability with an area under the receiver operating characteristic curve (AUC) of 0.864. To systematically analyze the possible biological functions of these genes, we conducted pathway enrichment analysis, which comprehensively revealed the potential molecular processes and signaling pathways they were involved in, thereby providing a solid theoretical basis and direction guidance for subsequent mechanism research. Furthermore, hierarchical clustering based on the expression profiles of these six genes enabled stratification of atherosclerotic patients into two distinct molecular subtypes, with subtype B exhibiting a significant association with plaque instability. These findings may offer novel insights for refining clinical diagnostic approaches and therapeutic strategies in atherosclerosis management.

Efferocytosis is a crucial physiological process by which the body eliminates apoptotic cells (Adkar and Leeper, 2024). It proceeds through three functionally discrete and temporally coordinated stages: recruitment (find-me), recognition (eat-me), and engulfment (Tajbakhsh et al., 2019). Among the 6 core EFRGs identified in this study, STAB1 (Stable Protein-1) is a scavenger receptor with immunomodulatory functions. It has been proven to regulate inflammatory responses and tissue homeostasis through multiple mechanisms. Phosphatidylserine (PS) is currently the most intensively studied and evidence-based “eat-me” signaling molecule (Leventis and Grinstein, 2010). There is clear experimental evidence indicating that STAB1 can directly bind to PS through its epidermal growth factor-like repeat domain (EGF-like repeats, EGFrp), thereby mediating the recognition and clearance of apoptotic cells by macrophages (Xiang et al., 2026). Notably, in the atherosclerosis animal model, monoclonal antibody inhibition of Stab1 significantly reduced Western diet-related atherosclerosis in ApoE-KO and Ldlr-KO mice (Manta et al., 2022). GULP1 (Protein with PTB domain 1) is a crucial efferocytosis adaptor protein. Studies have shown that the dysfunction of GULP1 can lead to impaired clearance of apoptotic cells, thereby driving chronic inflammation and tissue damage. Its abnormal expression or mutation has been confirmed to be associated with various diseases such as atherosclerosis, Niemann-Pick C disease, Alzheimer’s disease, rheumatoid arthritis, and schizophrenia (Chen et al., 2021). The CAMK2G gene encodes calcium/calmodulin-dependent protein kinase II gamma (CaMKIIγ). Previous studies have shown that inhibiting the activity or expression of CaMKIIγ can significantly increase the protein level of the phagocytic receptor MerTK in plaque macrophages, thereby enhancing the ability to clear apoptotic cells and improving plaque stability (Gao et al., 2024). During the process of efferocytosis, apoptotic cells need to undergo membrane lipid rearrangement mediated by phospholipid scramblase to flip phosphatidylserine (PS) from the inner to the outer surface of the plasma membrane, thereby exposing the “eat-me” signal to initiate clearance. ANO5 (anoctamin-5) is a calcium-activated phospholipid scramblase whose function has been confirmed to be involved in the dynamic regulation of cell membrane asymmetry. However, there is currently no direct experimental evidence indicating that ANO5 regulates phosphatidylserine (PS) flipping or affects efferocytic efficiency (Schmaier et al., 2023). LGR6 (leucine-rich repeat-containing G protein-coupled receptor 6) is a multifunctional G protein-coupled receptor. Recent studies have revealed that it can serve as a functional receptor for the pro-apoptotic lipid mediator maresin-1 (MaR1); through the MaR1–LGR6 signaling axis, it significantly enhances the ability of macrophages to clear apoptotic cells (Chiang et al., 2019). SCARF1 (Scavenger Receptor Family Member 1) is a highly conserved type-I transmembrane protein in evolution. When cells undergo apoptosis, the surface-exposed phosphatidylserine (PS) on its surface will bind to the complement C1q molecule. SCARF1 can specifically recognize and capture this “C1q-PS complex,” thereby accurately identifying apoptotic cells (Wicker-Planquart et al., 2020). In atherosclerosis, LGR6 and ANO5 lack direct causal evidence; in contrast, SCARF1 is established to mediate endocytosis of chemically modified lipoproteins—not efferocytosis—and contributes functionally to plaque development and stability. Future studies will test whether these genes influence plaque progression via efferocytosis regulation (Apostolov et al., 2009).

This study has several important limitations that merit careful interpretation. First, the analyses are inherently observational and computational; while our integrative bioinformatic approach yielded statistically robust and cross-dataset reproducible associations, it cannot establish causality without experimental confirmation. Second, although we employed statistical correction methods, the inherent biological and technical differences across the original studies may still affect the model’s generalizability and interpretability. Third, the absence of prospective clinical validation in independent patient cohorts constrains immediate translational utility and biomarker readiness. Collectively, these considerations underscore that our findings constitute hypothesis-generating insights rather than definitive conclusions—and thus necessitate rigorous downstream validation. Specifically, causal inference requires functional interrogation: genetic perturbation (e.g., CRISPR-Cas9 knockout or inducible overexpression in relevant vascular cell types), quantitative measurement of efferocytosis capacity in primary human macrophages or engineered co-culture systems, and longitudinal *in vivo* assessment of plaque morphology, composition, and stability in preclinical models of atherosclerosis. In summary, this study contributes novel insights into the molecular mechanisms of atherosclerosis from the perspective of efferocytosis regulation. The identification of six core biomarkers provides a foundation for the development of innovative diagnostic tools and targeted therapeutic strategies. Our results emphasize the promise of personalized medicine approaches in atherosclerosis management, particularly in enabling patient stratification based on efferocytosis-related gene expression profiles. Future research should focus on validating these biomarkers in clinical cohorts and elucidating their functional roles in disease progression, thereby advancing both mechanistic understanding and clinical outcomes in atherosclerosis.

## Data Availability

The original contributions presented in the study are included in the article/supplementary material, further inquiries can be directed to the corresponding authors.

## Nomenclature

AS: Atherosclerosis
DEGs: Differentially expressed genes
AS-EFRGs: Efferocytosis-related genes associate with atherosclerosis
EFRGs: Efferocytosis-related genes
ECs: Endothelial cells
GO: Gene ontology
GSEA: Gene set enrichment analysis
KEGG: Kyoto encyclopedia of genes and genomes
LASSO: Least absolute shrinkage and selection operator
NKT: Natural killer T
PPI: Protein-protein interaction
RF: Random forest
ROC: Receiver operating characteristic
scRNA-seq: Single-cell RNA sequencing
ssGSEA: Single-sample GSEA
SVM: Support vector machine
VSMCs: Vascular smooth muscle cells
WGCNA: Weighted gene co-expression network analysis
